# A high protein moderate carbohydrate diet fed at discrete meals reduces early progression of N-methyl-N-nitrosourea-induced breast tumorigenesis in rats

**DOI:** 10.1186/1743-7075-7-1

**Published:** 2010-01-10

**Authors:** Christopher J Moulton, Rudy J Valentine, Donald K Layman, Suzanne Devkota, Keith W Singletary, Matthew A Wallig, Sharon M Donovan

**Affiliations:** 1 Division of Nutritional Sciences, University of Illinois at Urbana-Champaign, 905 S Goodwin Ave, Urbana, IL 61801, USA; 2 Department of Kinesiology and Community Health, University of Illinois at Urbana-Champaign, 117 Louise Freer Hall, 906 S Goodwin Ave, Urbana, IL 61801, USA; 3 Department of Food Science and Human Nutrition, University of Illinois at Urbana-Champaign, 905 S Goodwin Ave, Urbana, IL 61801, USA; 4 Department of Veterinary Pathobiology, University of Illinois at Urbana-Champaign, 2522 VMBSB, 2001 S Lincoln Ave, Urbana, IL 61801, USA

## Abstract

Breast cancer is the most prevalent cancer in American women. Dietary factors are thought to have a strong influence on breast cancer incidence. This study utilized a meal-feeding protocol with female Sprague-Dawley rats to evaluate effects of two ratios of carbohydrate:protein on promotion and early progression of breast tissue carcinomas. Mammary tumors were induced by N-methyl-N-nitrosourea (MNU) at 52 d of age. Post-induction, animals were assigned to consume either a low protein high carbohydrate diet (LPHC; 15% and 60% of energy, respectively) or a high protein moderate carbohydrate diet (HPMC; 35% and 40% of energy, respectively) for 10 wk. Animals were fed 3 meals/day to mimic human absorption and metabolism patterns. The rate of palpable tumor incidence was reduced in HPMC relative to LPHC (12.9 ± 1.4%/wk vs. 18.2 ± 1.3%/wk). At 3 wk, post-prandial serum insulin was larger in the LPHC relative to HPMC (+136.4 ± 33.1 pmol/L vs. +38.1 ± 23.4 pmol/L), while at 10 wk there was a trend for post-prandial IGF-I to be increased in HPMC (*P *= 0.055). There were no differences in tumor latency, tumor surface area, or cumulative tumor mass between diet groups. The present study provides evidence that reducing the dietary carbohydrate:protein ratio attenuates the development of mammary tumors. These findings are consistent with reduced post-prandial insulin release potentially diminishing the proliferative environment required for breast cancer tumors to progress.

## Background

Breast cancer is the most common cancer in American women, with over 40,000 annual deaths and nearly 200,000 estimated new cases in 2009 [1]. Given the high prevalence of breast cancer and associated physical, social, and psychological detriments [2], reducing cancer incidence through modifiable risk factors such as nutrition is a high public health priority. Estimates suggest that cancer incidence could be reduced by ~1/3 through modifications in diet, including changes in total energy intake and diet content of fat, protein or carbohydrate [3]. With increased attention on higher protein, low carbohydrate diets for weight management, secondary effects of these diets on tumor development are important to evaluate.

Some studies report that dietary protein, and particularly consumption of animal protein, is linked to increased risk of breast cancer [4,5]. However, recent large-scale evaluations have failed to identify intakes of meat, eggs, or dairy products as risk factors [6]. Conversely, epidemiological studies have reported protective effects of dietary protein on breast cancer incidence [7] and mortality [8]. A recent case-controlled study suggests that a dietary pattern including the highest relative levels of animal protein is inversely associated with breast cancer risk [9].

Evidence is now accumulating that dietary carbohydrates, glycemic load, and hyperinsulinemia are associated with increased cancer risk [10,11] and mortality [12]. Higher levels of circulating and post-prandial insulin have been reported to enhance cellular proliferation and tumor development [13] through upregulation of the PI3K/Akt signal pathway which is implicated in breast cancer promotion and tumorigenesis [14]. Furthermore, insulin stimulates the ovaries to produce androgens [15], which have been linked to increased breast cancer risk [16]. To our knowledge, no studies have attempted to compare the effects of dietary protein versus carbohydrates in an experimental model of breast cancer.

We have previously demonstrated that feeding rats diets with increased protein and reduced carbohydrates enhances muscle insulin signaling and glucose uptake, attenuates post-prandial hyperinsulinemia, and stimulates skeletal muscle protein synthesis [17,18]. Our experimental model uses a meal-feeding protocol with three discrete meals each day to mimic human eating patterns and allow for isolation of post-prandial meal responses. This study examined the effects of feeding diets differing in ratios of carbohydrate/protein, but within the Dietary Reference Intake (DRI) acceptable macronutrient distribution range, on tumor incidence, size, and development, in a chemically-induced model of breast cancer. We hypothesized that a diet with a reduced carbohydrate:protein ratio would have a favorable impact on breast cancer development by tempering post-prandial insulin response.

## Methods

### Experimental Model

Female 45 d old Sprague-Dawley rats (n = 74; Harlan-Teklad, Indianapolis, IN) with a mean body weight of 135 ± 4.8 g were housed individually in stainless steel wire-bottomed cages and maintained at 24°C with 12 h reverse light cycle (light: 1900-0700 h) and free access to water. Rats were trained to consume three meals each day using a modified AIN-93G diet [19] (60% of energy from carbohydrate, 15% protein, and 25% fat; LPHC) as the baseline control. The meal pattern consisted of a 3 g breakfast meal (20% of total energy) consumed between 0700-0720 h, followed by free access to food between 1300-1400 h (~40% total energy) and 1800-1900 h (~40% total energy). At 52 d of age, animals were randomly assigned to consume either the baseline low protein high carbohydrate diet (LPHC; n = 37) or a high protein moderate carbohydrate diet (40% carbohydrate, 35% protein, and 25% fat; HPMC; n = 37) (Table 1). Both diet treatments were isoenergetic. Tumors were induced in all animals at 52 d of age by a single intraperitoneal dose of N-methyl-N-nitrosourea (MNU) at 50 mg/kg body weight in 1 ml of 0.9% NaCl [20]. Rats were weighed and food intake measured each week. The animal protocol was approved by the University of Illinois Institutional Animal Care and Use Committee.

**Table 1:** Diet Composition for Treatment Groups

Diet Component	LPHC	HPMC
Corn Starch	396.69	264.46
Sucrose	99.96	66.64
Maltodextrin	132.02	88.01
Casein	154.85	361.27
Soybean Oil	116.24	116.24
t-Butylhydroquinone	0.014	0.014
Choline	2.5	2.5
AIN93G Minerals	35	35
AIN93G Vitamins	10	10
Cellulose	50	50
Cystine	2.36	2.36

Grams per kg diet. LPHC and HPMC represent low protein and high protein diet groups, respectively.

### Measurements

At 3 wk post-MNU treatment, rats were randomly selected from LPHC (n = 12) and HPMC (n = 12) groups and evaluated for metabolic changes associated with diet treatments prior to tumor appearance. Rats were euthanized either after overnight food deprivation (fasted) or 90 min following consumption of a 3 g breakfast meal. Trunk blood was collected in K_3_EDTA vacutainers (BD, Franklin Lakes, NJ), placed on ice, and subsequently centrifuged at 1500 × G at 4°C for 20 min. Plasma was decanted and stored at -80°C for further analysis. Soleus and gastrocnemius muscles of the left leg were rapidly excised and frozen in liquid nitrogen, while the soleus and gastrocnemius of the right leg were dissected and weighed. Likewise, the left retroperitoneal fat pad was excised and frozen and the right retroperitoneal fat pad dissected and weighed. Liver was excised, weighed, and frozen. The 3 wk time point was intended to capture metabolic differences that could affect tumor promotion, while avoiding confounding pathology associated with tumor development.

At 10 wk post-induction, rats from LPHC (n = 12) and HPMC (n = 12) were measured for body fat using dual-energy X-ray absorptiometery (DXA) (Hologic, Inc., Bedford, MA). To isolate diet effects on body composition, only rats without palpable tumors were scanned. Rats were sedated with medetomidine hydrochloride (Orion, Espoo, Finland) at 0.3 mg/kg body weight 10 min prior to scanning, and were revived with atipamezole (Orion, Espoo, Finland) at 0.15 mg/kg body weight following the scan. All rats (LPHC: n = 25; HPMC: n = 25) were euthanized in a similar manner 3-4 d after DXA scanning and blood, soleus and gastrocnemius muscles, adipose, and liver were collected and weighed as previously described. Mammary tumors were rapidly excised, weighed, rinsed in saline, and a portion was fixed for 24 hr in 10% neutral buffered formalin for histological analysis. Fixed tumors were stored in 75% ethanol, then subsequently dehydrated and imbedded in paraffin. Imbedded tumors were then sectioned at 2-4 μM and stained with hematoxylin and eosin. Tumor sections were graded histologically on the following semi-quantitative scale: 0 - low grade adenocarcinoma, 1 - high grade adenocarcinoma, 2 - mild mammary hyperplasia, 3 - marked mammary hyperplasia.

### Carcinogenesis Parameters

Animals were monitored for mammary tumor appearance beginning at 5 wk post-induction through the remainder of the study and after animal euthanasia. Rats were palpated for mammary tumors 2 times/wk. When a tumor was detected, the date and tumor location were recorded. Tumor incidence and latency to tumor appearance were determined qualitatively by palpation. Tumor dimensions of length and width were measured orthogonally by caliper. Tumor surface area was calculated by the following formula: 4π × (length/2) × (width/2). Following euthanasia, all tumors were excised and weighed.

### Metabolic Analyses

Plasma glucose was measured by glucose oxidase assay (Thermo, Waltham, MA). Plasma insulin was measured by commercial RIA kit (Millipore, Billerica, MA). Plasma IGF-I was measured by RIA after dissociation from IGFBP by Sephadex G-50 chromatography in 0.2 M formic acid. Eluant containing the free IGF was collected and lyophilized (Labconco Freeze Dry Systems, Kansas City, MO). IGF-I concentrations were measured using [^125^I]-IGF-I as a competitive radioligand and a polyclonal anti-human antibody (National Hormone and Pituitary Program, NIDDK, Torrance, CA). Bound radioactivity was measured using a gamma counter (Packard Instruments, Meriden, CT) and concentrations were determined relative to a standard curve prepared with recombinant human IGF-I [21].

### Statistical Analysis

Data are expressed as mean ± SEM. Plasma glucose, insulin, and IGF-I were analyzed using 2-way ANOVA and between-diet differences were evaluated at each time point. Tumor incidence and time of appearance were evaluated using linear regression analysis. Histological classification of excised tumors was analyzed with a Mann-Whitney U test. All other carcinogenesis parameters, as well as body weight and food intake were analyzed by a Student's *t*-test. The level of significance was set at *P *< 0.05. All analyses were performed using SPSS Version 15.0 (Chicago, IL).

## Results

### Animal Weight Gain, Food Intake, and Body Composition

**Figure 1:**
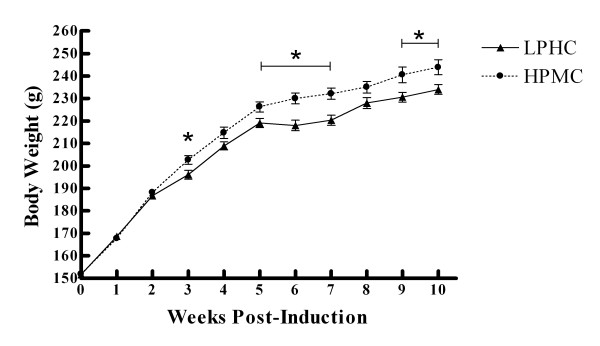
**Body Weights; Values represent Means ± SEM**. * indicates significant difference (P > 0.05).

Body weights of HPMC and LPHC groups were not different at baseline (Figure 1). At 3 wk, there were no differences between groups in body weight or weights of gastrocnemius, soleus, or retroperitoneal fat pad (Table 2). Body weight was greater in the HPMC group at wks 3, 5-7, and 9-10 (*P *< 0.05) (Figure 1). There were no differences in food intake between groups at any time during the study. At 10 wk, there were no differences in gastrocnemius or soleus weights between diet groups (Table 2) or in % body fat measured by DXA. However, retroperitoneal fat and liver were heavier in the HPMC group.

**Table 2:** Body Composition Measures

		LPHC	HPMC	*P *value
Gastrocnemius weight (g)	3 wk	1.32 ± 0.03	1.32 ± 0.05	0.939
	
	10 wk	1.54 ± 0.02	1.57 ± 0.03	0.398

Soleus weight (g)	3 wk	0.245 ± 0.006	0.237 ± 0.009	0.472
	
	10 wk	0.299 ± 0.007	0.309 ± 0.006	0.281

Adipose weight (g)	3 wk	0.374 ± 0.056	0.358 ± 0.055	0.844
	
	10 wk	0.443 ± 0.037	0.585 ± 0.047	0.021

Liver weight (g)	3 wk	-	-	-
	
	10 wk	5.60 ± 0.13	6.04 ± 0.11	0.012

% Body fat	10 wk	10.8 ± 0.6	11.2 ± 0.7	0.588

Values represent Mean ± SEM, n = 6 for each fasted and fed group.

### Serum Glucose, Insulin, & IGF-I Concentrations

Serum glucose was not different between groups at 3 or 10 wk (Table 3) in either fasted or post-prandial blood samples. At 3 wk, fasting insulin tended to be higher in the HPMC group (*P *= 0.09), while the meal response of serum insulin was greater in the LPHC group compared to HPMC group (+136.4 ± 33.1 pmol/L vs. +38.1 ± 23.4 pmol/L, respectively; *P *= 0.035). At 10 wk, post-prandial serum insulin was elevated in both LPHC and HPMC groups compared to fasted animals. Serum IGF-I was not significantly modified by diet or meal-feeding at 3 wk. At 10 wk there was a trend for serum IGF-I to be increased post-prandially in the HPMC group (*P *= 0.055).

**Table 3:** Plasma Metabolic Measurements

			Fasted	Fed	Time Effect *P *Value	Time × Diet Effect *P *Value
Glucose (mmol/L)	3 wk	LPHC	6.86 ± 0.50	6.74 ± 0.44	0.854	0.841
		HPMC	7.16 ± 0.48	6.84 ± 0.47	0.642	
	
	10 wk	LPHC	6.73 ± 0.18	6.54 ± 0.27	0.565	0.656
		HPMC	6.70 ± 0.27	6.74 ± 0.31	0.917	

Insulin (pmol/L)	3 wk	LPHC	77.4 ± 11.6	213.8 ± 33.1	0.003	0.075
		HPMC	125.1 ± 19.8	163.2 ± 23.4	0.350	
	
	10 wk	LPHC	86.2 ± 10.4	194.4 ± 29.7	0.002	0.520
		HPMC	77.5 ± 5.8	220.2 ± 37.6	0.003	

IGF-I (μg/L)	3 wk	LPHC	240 ± 24	272 ± 26	0.396	0.564
		HPMC	274 ± 10	268 ± 32	0.917	
	
	10 wk	LPHC	205 ± 30	211 ± 17	0.851	0.221
		HPMC	237 ± 17	299 ± 23	0.055	

Values represent Mean ± SEM, n = 6 for each fasted and fed group

### Carcinogenesis Parameters

**Figure 2:**
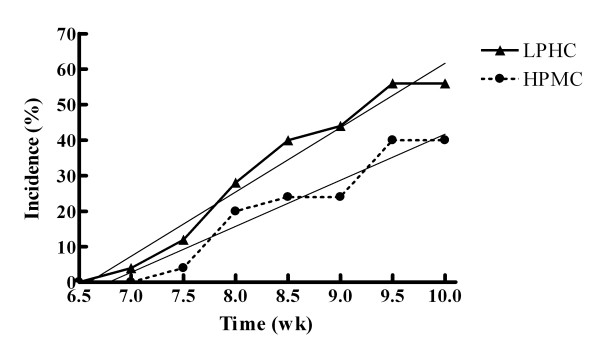
**Tumor Incidence; Comparison of slopes of linear regression (P = 0.019); LPHC: y = 18.10x - 119.3, r^2 ^= 0.968; HPMC: y = 12.95x - 87.86, r^2 ^= 0.937**.

At 3 wk post-induction, no animals had palpable tumors and no tumors were found during dissection. Initial palpable tumors were detected at 51 d in the LPHC group and 55 d in the HPMC group. Surface area of palpable tumors was not different between treatments at any time point. The rate of tumor incidence was higher in the LPHC group with relative slopes of tumor incidence of 18.2 ± 1.3%/wk and 12.9 ± 1.4%/wk (*P *< 0.05) for the LPHC and HPMC groups, respectively (Figure 2). Other characteristics of tumor incidence, tumor latency (average time to first tumor development), and cumulative tumor mass at 10 wk, were not different between treatments (Table 4). All excised tumors were confirmed histologically to be mammary ductal carcinomas, and there were no differences between groups in the grade of the carcinomas.

**Table 4:** Carcinogenesis Parameters

	LPHC	HPMC	*P *value
Tumor Incidence at 10 wk	0.56	0.4	> 0.05
Tumor Latency (wk)	8.36 ± 0.21	8.60 ± 0.26	> 0.05
Cumulative Tumor Mass (g)	2.22 ± 0.67	2.51 ± 1.40	> 0.05
Rate of Tumor Incidence (% per wk)	18.2 ± 1.3	12.9 ± 1.4	0.019

Values represent Means ± SEM.

## Discussion

The objective of this study was to identify potential effects of manipulating the dietary carbohydrate:protein ratio on the promotion of MNU-induced breast cancer tumors. A major finding of our study is that a HPMC diet with an reduced carbohydrate:protein ratio significantly reduced the rate of incidence of palpable mammary tumors. Although tumor latency was not different between groups, once palpable tumors began to appear, the rate of tumor appearance proceeded more rapidly in the LPHC group. This suggests that either reduced dietary carbohydrate or elevated dietary protein (or both), can attenuate the early development of mammary tumors. In agreement with our previous study [17], post-prandial insulin at 3 wk was elevated in the LPHC group and not in the HPMC group. Thus, animals in the LPHC group were likely exposed to higher, repeated elevations in serum insulin following each discrete meal during the period leading to the emergence of palpable mammary tumors. Post-prandial insulin at 10 wk was elevated in both groups, although host metabolism was likely confounded by tumor pathology, and this change does not necessarily diminish the early effect of post-prandial insulin on tumor promotion.

Nearly two decades ago, hyperinsulinemia was identified as a significant risk factor for breast cancer, independent of body composition or adiposity [22]. Cohort studies have found that women surgically treated for breast cancer with high fasted insulin levels had substantially increased risk for reoccurrence and death [23] and that the highest tertile of fasted insulin levels was associated with a 2-fold increase in post-menopausal breast cancer risk [24,25]. Most recently, the presence of metabolic syndrome was reported to be associated with breast cancer risk [26], and insulin resistance and post-prandial hyperinsulinemia are central features of metabolic syndrome [27], leading to suggestions that metabolic syndrome may serve as a prognostic factor for breast cancer [28]. With respect to dietary factors, a positive association between high glycemic index/load and breast cancer risk has been identified [29,30]. High total carbohydrate intake [31] and sucrose intake have been associated with unfavorable outcomes for breast cancer.

One of the mechanisms by which insulin has been proposed to increase breast cancer risk is via IGF-I. Elevated insulin can increase serum free IGF-I concentrations [32]. Overexpression of IGF-I in mammary epithelium *in vivo *leads to hyperplasia, spontaneous tumorogenesis, and enhanced susceptibility to chemical carcinogens [33], and circulating IGF-I appears to be important for the onset and subsequent development of mammary tumorigenesis in *in vivo *experiments [34,35]. In the present study, the elevated post-prandial insulin observed in the LPHC group was not associated with increased serum IGF-I concentrations.

Clinically, the effect of IGF-I on breast cancer remains equivocal. Elevated serum IGF-I concentrations appear to increase risk of pre-, but not post-menopausal breast cancer, while higher blood glucose levels were associated with increased risk for developing post-menopausal breast cancer [36]. However, a case control study identified a weak association for IGF-I with breast cancer in women under the age of fifty [37]. Most recently, the Women's Health Initiative study found a strong association between fasting insulin levels and breast cancer in post-menopausal women, but no association of free IGF-I [38].

IGF-I bioactivity is modulated by IGF binding proteins (IGFBP), which can block IGF-I from binding to its receptor. At least 75% of circulating IGF-I is bound to IGFBP-3 [39], though IGFBP-3 levels remain fairly constant within individuals [40]. IGFBP-1 and IGFBP-2 levels are inversely associated with insulin levels [41], which suggests that chronic hyperinsulinemia could depress binding protein interaction and thus increase free IGF-I. Serum IGF-I levels have also been shown to relate to protein intake [42]. In the present study at 10 wk, there was a trend for post-prandial IGF-I to be higher (p = 0.055) in the HPMC group. Despite the elevation of IGF-I in the HPMC group, the rate of tumor incidence was significantly lower.

Consistent with our findings, early animal experiments investigating the influence of dietary protein on breast cancer found that feeding a higher protein diet prior to administration of a carcinogen resulted in a reduction of tumor prevalence [43], suggesting a protective effect of protein during the initiation phase. Additionally, increased dietary protein fed after tumor induction had no independent effect on the promotion phase of carcinogenesis [44]. In contrast to the findings of the present study, Hawrylewicz et al. found higher dietary protein (33% vs. 15%) increased the number of tumors and tumor weight in MNU-induced rats, despite no difference in tumor incidence or latency [45]. However, this study differed substantially from the current study in that the rats were exposed in utero from dams fed the test diets and then consumed the same test diets *ad libitum *after weaning.

A further consideration is that elevated dietary protein was accomplished at the expense of dietary carbohydrate, and since tumors are obligate glucose consumers, HPMC may have been less energetically favorable to neoplastic tissues. Similarly, a ketogenic diet (80% medium chain triglycerides) fed to mice with implanted colon adenocarcinomas reduced tumor weight, while the inclusion of hydroxybutyrate in drinking water counteracted the stimulation of tumor growth by insulin infusion [46]. However, fasted glucose was not different between groups at either 3 or 10 wks, and in reflecting current dietary guidelines, the HPMC diet was not low enough in carbohydrate to induce ketogenesis.

A limitation of the current study is its relatively short length. At 10 wks, tumor incidence had only reached 40% in the HPMC group. Due to the labor-intensive nature of the meal-feeding protocol, this study was designed for 10 wk, with a goal of both groups exceeding 50% tumor incidence. However, within the short period of time, the study still achieved divergent rates of tumor appearance between diet groups.

## Conclusion

In total, the present study provides evidence that reducing the dietary carbohydrate:protein ratio attenuates the progression of mammary tumors, and this finding is consistent with reduced post-prandial insulin release potentially diminishing the proliferative environment required for breast cancer tumors to progress. These results warrant additional investigation into the potentially protective effect that elevated dietary protein and reduced carbohydrate may have on breast cancer development.

## Competing interests

The authors declare that they have no competing interests.

## Authors' contributions

CM participated in the design of the study, coordinated the study, performed the statistical analysis, and drafted the manuscript. RV assisted in performing the study and helped to draft the manuscript. DL conceived of the study, participated in its design, and helped to draft the manuscript. SD helped in conception of the study and its design. KS helped in the conception of the study and its design. MW performed the histochemical analysis. SMD helped to draft the manuscript. All authors read and approved the final manuscript.
